# Seasonal Changes in Essential Oil Constituents of *Cystoseira compressa*: First Report

**DOI:** 10.3390/molecules26216649

**Published:** 2021-11-02

**Authors:** Ivana Generalić Mekinić, Martina Čagalj, Giulia Tabanelli, Chiara Montanari, Federica Barbieri, Danijela Skroza, Vida Šimat

**Affiliations:** 1 Department of Food Technology and Biotechnology, Faculty of Chemistry and Technology, University of Split, R. Boškovića 35, HR-21000 Split, Croatia; gene@ktf-split.hr (I.G.M.); danci@ktf-split.hr (D.S.); 2 University Department of Marine Studies, University of Split, R. Boškovića 37, HR-21000 Split, Croatia; martina.cagalj@unist.hr; 3 Department of Agricultural and Food Sciences, University of Bologna, Viale Fanin 44, 40127 Bologna, Italy; giulia.tabanelli2@unibo.it; 4 Department of Agricultural and Food Sciences, University of Bologna, Piazza Goidanich, 47521 Cesena, Italy; chiara.montanari8@unibo.it (C.M.); federica.barbieri16@unibo.it (F.B.)

**Keywords:** *Cystoseira compressa*, brown algae, essential oil, harvest period, GC–MS

## Abstract

Marine macroalgae are well known to release a wide spectrum of volatile organic components, the release of which is affected by environmental factors. This paper aimed to identify the essential oil (EO) compounds of the brown algae *Cystoseira compressa* collected in the Adriatic Sea monthly, from May until August. EOs were isolated by hydrodistillation using a Clavenger-type apparatus and analyzed by gas chromatography coupled with mass spectrometry (GC–MS). One hundred four compounds were identified in the volatile fraction of *C. compressa*, accounting for 84.37–89.43% of the total oil. Samples from May, June, and July were characterized by a high share of fatty acids (56, 69, and 34% respectively) with palmitic acid being the dominant one, while in the August sample, a high content of alcohols (mainly phytol and oleyl alcohol) was found. Changes in the other minor components, which could be important for the overall aroma and biological activities of the algal samples, have also been noted during the vegetation periods. The results of this paper contribute to studies of algal EOs and present the first report on *C. compressa* EOs.

## 1. Introduction

More than 70% of the Earth’s surface is covered with oceans and seas, so it is not surprising that marine ecosystems are extremely complex with tremendous biodiversity. Recently, there is a growing trend in the investigation of new, inexpensive, and valuable sources of biologically active compounds, and marine origin products, like algae, are one of the most interesting sources, due to their production of a great variety of unique secondary metabolites [1]. Algae are vegetative organisms widely distributed throughout the world. Although many of them are of commercial importance in some parts of the world due to their nutritional, biological, and functional properties, only a small number of species are currently exploited for industrial food applications [2]. Studies on marine algae are usually focused on the isolation of structurally different bioactive compounds like polysaccharides (e.g., fucoidan, alginate, and laminarin), photosynthetic pigments (carotenoids, chlorophylls, and phycobilins), sterols, polyphenolics, etc. [3,4,5,6,7,8,9]. In comparison to the research on these non-volatile compounds, studies on volatiles of marine origin are still scarce.

Essential oils (EOs), as a special chemical group of algal metabolites, play an important role in communication in marine ecosystems, both interspecies and intraspecies, as well as in interactions with the surrounding environment. These compounds are involved in various algal ecological functions: they are defenses against predators and herbivores; they act as pheromones (allelochemicals; take part in the adaptation to abiotic stresses; and are important for the inhibition of bacterial and/or fungal fouling [1,10,11,12]. The essential oil metabolites present in marine algae species contain a mixture of different chemical classes such as hydrocarbons, fatty acids, esters, alcohols, carboxylic acids, aldehydes, ketones, terpenes, polyphenols, furans, pyrazines, pyridines, halogenated amines, and sulphur compounds [1,2]. The production of algal EOs is closely related to the physiology of the species [11,12]. Studies on EOs of green and red algae mainly report the presence of monoterpenoids, halogenated compounds, and sulphur compounds that have a low impact on their aroma perception. In contrast to those species, brown algae is responsible for strong and pleasant marine odors (the so-called “beach note”), which is usually related to the presence of C11-hydrocarbons. Among other aroma compounds, these species contain a wide range of monoterpenoids and sesquiterpenoids [13]. Although the functions of algal EOs are similar to those in terrestrial plants, studies dealing with algal EOs and their role are still in the primary stage, and there is a lack of reports on this subject [12]. EO profiles differ between species, but they are also influenced by various factors as age, geographical origin, growth and nutrition conditions, season, temperature, light, salinity, and processing/extraction parameters [2,12].

There are about 40 species of algae from the genera *Cystoseira* (Phaeophyta), which are widely distributed along the Eastern Atlantic and Mediterranean coasts [14], and *C. compressa*, is one of the most widespread brown algae in the Adriatic Sea. *C. compressa* is attached to the substratum by a small disc and its thallus shows morphological plasticity. Changes are most evident in the spring/summer period, when the winter rosette shape of the branches shifts to dense and ramified branches with aerocysts [15]. These changes might be related to the length of the photoperiod and sea temperature, and their effect on the EOs or other chemical components of the algae (phenolic profile, pigments, etc.) is unknown.

Compounds from *C. compressa* were characterized from extracts and associated with various biological activities, e.g., polysaccharides and phlorotannins with antioxidant activity [16,17], phlorotannins with antidiabetic activity [17], and phenolic compounds with antibacterial activity [5]. Furthermore, a connection between total phenolic content and the seawater temperature was observed, showing that the amount of phenolics is influenced by the temperature [18]. However, characterization of EO components has been done for *C. sedoides* [13], *C. barbata* [19,20], *C. crinita* [19], and *C. tamariscifolia* [21], but to our knowledge, there are no reports on compounds of *C. compressa* and their comparison over the spring/summer period, when the algae are under the influence of the thallus change, a rise in sea temperature, and an intensive photoperiod. For these reasons, this work aimed to study the EO profiles of *C. compressa*, collected in the Adriatic Sea monthly from May until August, to identify the molecules characterizing this species.

## 2. Results and Discussion

Seaweeds are widespread around the world, being of commercial importance in some parts, where they are consumed fresh, dry, or as an ingredient. Although in some regions they are widely used in the human diet, only a small number of species are currently exploited for food applications. One of the main limitations of the use of algal materials in the food industry is their flavor, which is the main parameter of quality directly related to consumers’ acceptance of food [2]. In comparison to the terrestrial odoriferous plants, only some algae possess an attractive, pleasant odor and characteristic marine flavor, and, therefore, great potential to be used in various food and cosmetic preparations [1,13].

Different extraction methods like hydrodistillation, solvent extraction, microwave-assisted extraction, supercritical fluid extraction, headspace extraction, etc., are commonly used for the isolation of volatile analytes from algal materials. In recent times, the conventional extraction procedures are usually being replaced by novel techniques that are less time-consuming, often (fully) automated, more environmentally friendly, require less solvent, and are more efficient [8]. However, despite all its disadvantages (duration, high temperatures, low efficiency, potential degradation of compounds, etc.), hydrodistillation is still the most used method. On the other hand, identification of the EO components is usually performed using capillary gas chromatography coupled with mass spectrometry (GC–MS), as this method of characterization covers a wide spectrum of compounds, from non-polar to polar ones [11,13].

**Figure 1:**
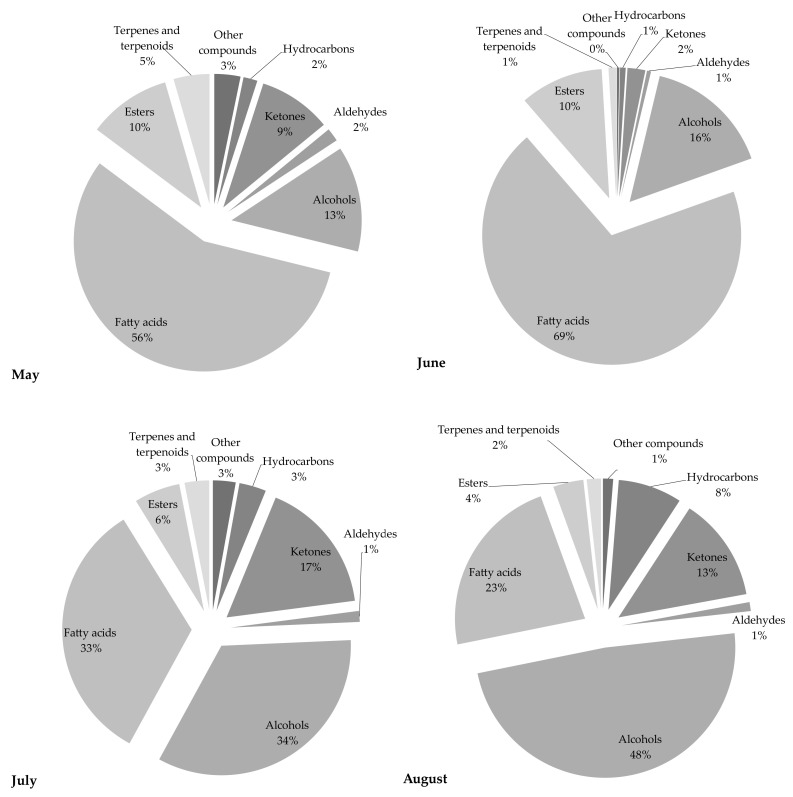
The relative content of each chemical class of compounds detected in *C. compressa* samples.

**Figure 2:**
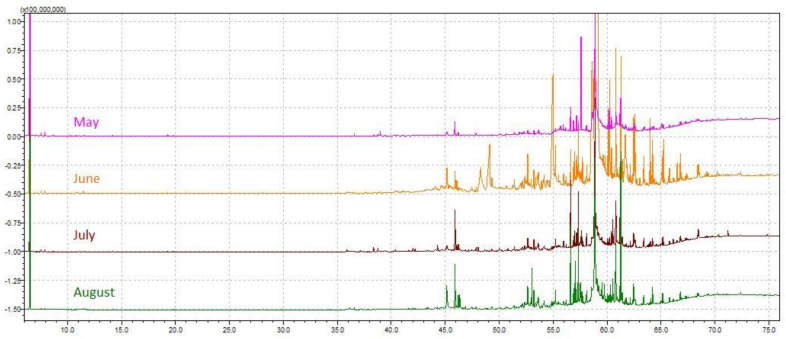
GC–MS chromatograms of the essential oils obtained from *Cystoseira compressa* collected from May to August.

The chemical profile of volatile fractions and the relative content of detected components obtained by hydrodistillation of *C. compressa* are reported in Table 1. One hundred four compounds were identified, accounting for 84–89% of the total chemical composition. Figure 1 presents the relative share of the sum of compounds from the same chemical class to get better insight into the algal EOs profile. The GC–MS chromatograms of the essential oils obtained from *C. compressa* collected in different months are shown in Figure 2.

Samples from May and June were characterized by a high share of fatty acids, while in the July and August samples the dominant chemical class of compounds were alcohols (34 and 48%, respectively). EOs from May and June were characterized by an extremely high content of fatty acids, 56 and 69%, respectively, while almost two-fold lower results were obtained for the July extract. The major acid in all samples was palmitic acid (C16:0), with the highest amount found in the May extract (40.15%), and shares of 31.92%, 26.81%, and 18.62%, in the June, July, and August samples, respectively. It is interesting to note that this saturated fatty acid was present in high amounts in all samples and followed a regular trend characterized by a continued decrease in content during the collecting months. In comparison to the May samples, there was a more than two-fold lower amount detected in the August samples. This compound was also previously reported as an abundant fatty acid in different *Cystoseira* species [3,4,8,14,21,22]. The May extract also contained the highest share of eicosanoic acid (2.58%). Significant amounts of this acid were also found in June (0.51%) and July (1.14%), while it was not detected in the August sample. The content of all other fatty acids was the highest in the June fraction: palmitoleic acid (11.94%) > myristic acid (7.60%) > lauric acid (3.78%) > (*Z*)-dodec-5-enoic acid (2.79%) > oleic acid (1.64%) > arachidonic acid (1.45%) > stearic acid (0.36%). It is well known that fatty acids with >12 carbon atoms are odorless, so although present in high amounts they do not affect significantly the flavor of the samples [2].

Among monounsaturated fatty acids, the presence of (*Z*)-5-dodecenoic acid was confirmed only in the June sample, where the content of oleic acid was also the highest in comparison with the others. Arachidonic acid was the only detected polyunsaturated acid, with the highest amount again found in the June sample, but significant amounts were also detected in May (0.96%). Cvitković et al. [8] reported the domination of total unsaturated fatty acids in the lipid fraction of different Adriatic brown algae species and two *Cystoseira* species, *C. barbata* and *C. compressa*. These authors also reported the domination of oleic acid among unsaturated fatty acids, as well as the presence of arachidonic acid in high amounts in brown algae samples. Similar results were also reported by Oucif et al. [4]. Kord et al. [22] also identified fatty acids (14 to 20 carbon atoms) of which palmitic acid was the major compound in *C. sauvageauana* lipid fractions, while among polyunsaturated fatty acids, arachidonic acid was the found in highest concentration.

Compounds from the chemical class of hydrocarbons, alkanes, and alkenes are common compounds in the majority of marine macroalgae EOs [1]. Although unsaturated hydrocarbons from C8 to C19 with the presence of 1 to 4 degrees of unsaturation are common, our study mainly reported the presence of compounds with one double bond. From the class of hydrocarbons, the straight chain saturated hydrocarbon 11-pentan-3-ylhenicosane was found in high amounts (from 0.43% to 1.41%), as well as hexadecane (from 0.08% to 1.30%). Both of these compounds followed similar trends, with the lowest concentrations found in the July sample, while their content significantly increased in next two collecting months, with the highest concentration in August. Also, pentadec-1-en was found in July (0.14%) and in even higher amounts in August (2.62%), while in the first two collection months this compound was not detected. The presence of squalene, which is the biosynthetic precursor of triterpenes and steroids, was confirmed in all samples, with the highest amounts detected in July.

Previous studies on volatile components from *Cystoseira* species confirmed the domination of hydrocarbons in *C. barbata*, while this class of compounds was found only in traces in *C. crinite*, where the majority of compounds were monoterpenoids [19]. The domination of hydrocarbons in the volatile oil of *C. barbata* was also reported by Ozdemir et al. [20], while Bouzidi et al. [13] reported that the most important class of VOCs obtained by hydrodistillation in *C. sedoides* were fatty acids and derivatives, with a content of 53.1%. Gressler et al. [11] reported the identification of hexadecane in different algae, among which were two *Cystoseira* species: *C. barbata* and *C. mediterranea*. Furthermore, heneicosan was also detected in *C. barbata* [20]. In their study, Bouzidi et al. [13] confirmed the presence of hexadecane and pentadec-1-en in samples of the Algerian endemic algae *C. sedoides*. It is interesting to note that these compounds were found in samples obtained by hydrodistillation, while they were not present in fractions obtained by focused microwave hydrodistillation and supercritical fluid extraction, which could be confirmation that aggressive isolation conditions (e.g. high temperature, long extraction duration, oxidation, and contact with water) cause the degradation of volatiles.

Samples from July and August contained high percentages of alcohols, 34% and 48%, respectively. Phytol, an acyclic diterpene alcohol, also known as a precursor of vitamin E and a degradation product of chlorophyll, was found in all samples at the highest percentage, especially in the August sample, where its content was 14.20% of all detected compounds [1]. This compound was detected in the lowest concentration in the June sample (2.9%), but in the next two months its content was almost 2 and 5-fold greater. El Amrani Zerrifi et al. [21] confirmed the domination of phytol in *C. tamariscifolia* from their study, as well as Bouzidi et al. [13] in *C. sedoides*. Other dominant components from the chemical class of alcohols were oleyl alcohol and *n*-nonadecan-1-ol, for which the regular amount increase during the collecting months was recorded. The presence of oleyl alcohol in the May sample was not confirmed, while its content in June was 0.68%, in July 5.76%, and in August almost 6%. On the other hand, the share of *n*-nonadecan-1-ol was 1.67% in May, 3.13% in June, 4.13% in July, and 4.34% in August, and an increase in its concentration during the collection periods could be noted. The great impact of unsaturated alcohols on the overall aroma and sensory perception of food has been previously reported [2].

The share of ketones was 9% in the May sample, 13% in the August sample and 17% in the July sample, while the lowest amount was found in the June sample (only 2%). Among detected compounds, (*E*)-4-(2,6,6-trimethyl-1-cyclohexen-1-yl)-3-buten-2-one (ranging from 0.53% to 5.41%) and 6,10,14-trimethyl-pentadecan-2-one (ranging from 0.75% to 5.98%), were found in the highest amounts. It is interesting to note that the amounts and the variations in their content among samples for both compounds followed the same trend: July > August (5.72% and 5.41%, respectively) > May (2.76% and 2.58%, respectively) > June. Bouzidi et al. [13] also reported the identification of 6,10,14-trimethyl-pentadecan-2-one in *C. sedoides.* Among other detected ketones, significant amounts of tridecan-2-one and dec-1-en-3-one were found. The first component was detected in the highest amount in the July sample (0.67%), while the other one was found in the May sample (0.42%). The July sample was also rich in monoterpene ketone geranyl acetone (0.70%).

Among all detected compounds, aldehydes, which are important odor compounds, were detected in the lowest percentages in all samples (1–2%), with only a few compounds present at a percentage above 0.10%. Aldehydes with low molecular weight are associated with unpleasant aroma, while those with higher molecular weight are responsible for sweet and fruity notes [2]. Tridecanal was dominant in all samples ranging from 0.37% in June to 0.81% in July. Tetradecanal was found in the highest amount in August (0.20%), while its presence in June was not confirmed. On the other hand, (*Z*)-undec-4-enal was found in the May sample at a percentage of 0.36%, while in other samples it was not detected.

The share of esters in the first two collecting months was equal (10%), while in July and August it was significantly lower, at 6% and 4%, respectively. The dominant ester was methyl arachidonate, with the highest amount found in the May sample (4%). Its content was significantly lower in June (2.49%), July (1.55%), and August (1.74%). Other benzoic acid esters were also found in high amounts in all samples, especially tetradecyl ester in the June sample (4.37%). The highest content of other esters, namely pentadecyl and tridecyl benzoate, were detected in samples harvested in June. Finally, it is interesting to note that all these compounds—tri, tetra, and penta-decyl esters—showed a similar trend across the collecting months: June > July > May > August.

Terpenes are a class of compounds that play an important role as chemical defense agents, but are also involved in some metabolic processes and functions, like the stability of cell membranes and photosynthesis [1]. It has been reported that terpenes are responsible for the distinctive ocean smell of algae, particularly acyclic and cyclic non-isoprenoid C11-hydrocarbons, while the disagreeable odor is related to amines and halogenated, sulphurous, and other specific compounds [1]. However, for the detection of polycyclic aromatic hydrocarbons, substituted phenols, and sulphur compounds, liquid chromatography is required, as they are semi-volatile [11]. From the group of terpenes, a terpene ketone farnesyl acetone (6,10,14-trimethylpentadeca-5,9,13-trien-2-one) was found in the highest amount in all samples (from 0.57% in June to 1.28% in July). The joint FAO/WHO Expert Committee on Food Additives put this compound on its list of flavoring agents, as it is characterized by an intensely sweet and floral odor, which makes it interesting for further applications [23]. Among others, alpha-cadinol was dominant in the May sample at 1.24%; its content was significantly lower in June, while in samples from other to collecting months it was not detected. Bouzidi et al. [13] also reported the presence of this compound in their study, though again, only in samples prepared by hydrodistillation.

Previous studies on *Cystoseira* species confirmed the potential health benefits of algae extracts and present individual compounds. Bruno de Sousa et al. (2017) in their review paper reported various biological activities of the *Cystoseira* algae samples, among which properties like antioxidant, antimicrobial (antibacterial, antifungal, antiviral), cytotoxic, antiproliferative, anticancer, antifouling, anti-inflammatory, antileishmanial, cholinesterase inhibitory, anti-diabetic, anti-obesity, hepatoprotective, etc. were confirmed by different studies.

Among recent studies, Hentati et al. [16] detected good antioxidant activity of water-soluble polysaccharides (fucoidan and a sodium alginate), while antidiabetic and antioxidant activity of phlorotannins extracted from *C. compressa* were reported by Gheda et al. [17]. Abu-Khudir et al. [24] investigated and confirmed the good free radical scavenging activity of the *C. crinita* extracts, antimicrobial activity against various pathogenic microorganisms, and strong cytotoxic effects against a panel of cancer cells. The authors, using GC–MS analysis, also confirmed the presence of a vast array of medicinally valuable phytochemical compounds belonging to various classes. Ahmed et al. [25] investigated the antimicrobial and cytotoxic activity of the extract, fractions, and pure compounds from *C. trinodis*, and their results pointed out the good activity of the samples.

Although the yield of EOs obtained from algal samples is low, *C. compressa* could be an interesting subject of further analysis on algae biological activities, due to the results of previous studies and the interesting chemical profiles of isolates (EOs and extracts from our other study).

## 3. Materials and Methods

### 3.1. Algal Material

**Figure 3:**
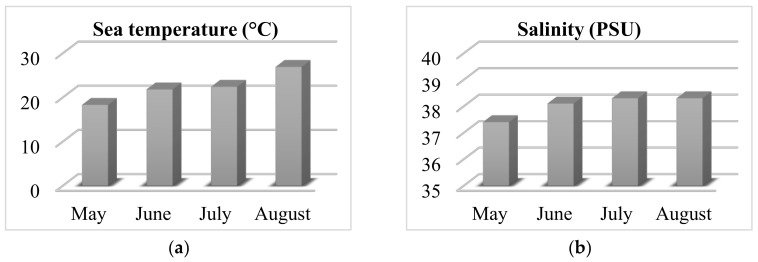
Sea parameters during the algal material sampling; (**a**) Temperature and (**b**) Salinity.

The wild-growing populations of *C. compressa* (Phaeophyceae) were collected monthly from May to August 2020 on the coast of Čiovo Island, Central Dalmatia, Croatia (43.493389° N, 16.272505° E). Samples were collected throughout a lagoon at 25 points in depth, ranging from 20 to 120 cm. During every sampling, the sea parameters (temperature in °C and salinity in Practical Salinity Unit, PSU) were measured using an YSI Pro2030 probe (YSI Inc., Yellow Springs, OH, USA) and the obtained results are shown in Figure 3. The sea temperature rose during the months of sampling, while the salinity changed under the influence of water springs (typical only in periods with sufficient rainfall, while in periods of drought the springs cease to flow). Pre-treatment of the algal material involved removal of sand, epiphytes, and other organisms from the surface by washing it with tap water. The algal materials were air-dried (for 7 days at room temperature in a shaded and aerated place) and dried algal materials were used for the isolation of the volatile organic compounds.

### 3.2. Extraction of Essential Oils

*C. compressa* essential oils were obtained by hydrodistillation of dried algal material (100 g) that was immersed in a flask with distilled water (1000 mL). The extraction process was performed in a Clavenger apparatus (Deotto Lab, Zagreb, Croatia) for 3 h. Pentane and diethyl ether (1:1, *v*/*v*) in the inner tube of the apparatus were used for trapping the volatile compounds carried through the system by vapor. Finally, after hydrodistillation, the distillate was dried over anhydrous sodium sulphate while nitrogen was used to evaporate the organic solvent. The samples of essential oils were stored at +4 °C in the dark until analysis [21,26,27].

### 3.3. GC–MS Analysis of Volatiles

The seaweed EOs were analyzed by GC–MS (Shimadzu QP2010, Shimadzu, Kyoto, Japan) using an autosampler and a DB-5 60 m × 0.25 mm × 0.25 μm column (Agilent Technologies Italia Spa, Milano, Italy). The EOs were resuspended in hexane and 1 µL was injected in the following gas chromatographic conditions: injection temperature 260 °C, interface temperature 280 °C, ion source 220 °C, carrier gas (He) flow rate 30 cm/s, splitting ratio 1:10. The oven temperature was programmed as follows: 40 °C for 4 min, from 40 °C to 175 °C with a 3 °C/min rate of increase, from 175 °C to 300 °C with a 7 °C/min increase, then holding for 10 min. EO constituents were identified by comparing their mass spectra with those reported in literature and the NIST Mass Spectral Database (NIST 08, National Institute of Standards and Technology, Gaithersburg, MD, USA). For each sample, the volatile profile composition was expressed as the relative percentage of each single peak area with respect to the total peak area.

## 4. Conclusions

This paper is the first report that provides information about the influence of the harvest period on essential oil aromatic compounds in *C. compressa*, and to obtain insight into the impact of individual components on the general sensory perception of the algae. According to the results obtained, *C. compressa* could be considered as a source of novel chemical entities with great potential to be used as an ingredient in different industrial applications such as functional foods, pharmaceuticals, and/or cosmeceuticals. The increase in the content of some of the key aroma compounds during the vegetation periods has been noted, while some detected compounds are probably products of degradation or modifications caused by aggressive isolation conditions. As new extraction methods have greatly developed in the last few years and have been widely used in the field of natural compounds due to their numerous benefits in comparison to conventional ones, this scientific research is still ongoing and opens a wide spectrum of possibilities for future research.

## Figures and Tables

**Table 1:** Differences in the chemical composition of essential oils of *C. compressa* harvested in different periods. The data are expressed as relative percentages of each single peak area with respect to the total peak area.

Compound							*C. compressa* Samples			
No.	Rt (min)	Name	Similarity (%)	MW	Formula	CAS	May	June	July	August
1	7.10	Pent-1-en-3-one	95	84.12	C_5_H_8_O	1629-58-9	0.07	n.d.	n.d.	n.d.
**2**	**7.55**	**Tetrahydro-2,5-dimethyl-furan**	**97**	**100.15**	**C_6_H_12_O**	**1003-38-9**	**0.88**	0.06	0.35	0.16
3	10.24	Tetrahydro-2-furanmethanol	92	102.13	C_5_H_10_O_2_	97-99-4	0.06	n.d.	0.02	0.01
4	10.85	Hex-5-enal	93	98.14	C_6_H_10_O	764-59-0	n.d.	0.01	0.01	0.01
5	11.07	Hexan-2-one	93	100.16	C_6_H_12_O	591-78-6	0.05	n.d.	0.03	0.01
6	11.33	Hexan-3-ol	90	102.17	C_6_H_14_O	623-37-0	0.07	n.d.	0.03	0.03
7	11.48	Hexanal	95	100.16	C_6_H_12_O	66-25-1	0.10	0.04	0.07	0.07
8	14.16	(*E*)-Hex-2-enal	91	98.14	C_6_H_10_O	6728-26-3	0.04	0.01	0.04	0.04
9	16.75	Heptanal	92	114.19	C_7_H_14_O	111-71-7	0.04	0.01	0.01	0.01
10	19.81	1-methyl-pentyl hydroperoxide	88	118.17	C_6_H_14_O_2_	24254-55-5	0.11	0.01	0.05	0.02
11	20.97	Oct-1-en-3-one	90	126.19	C_8_H_14_O	4312-99-6	0.03	0.02	0.05	0.01
12	24.17	Propylcyclohexane	82	126.24	C_9_H_18_	696-29-7	0.01	n.d.	0.02	n.d.
13	25.31	(*E*)-Oct-2-enal	90	126.20	C_8_H_14_O	2548-87-0	n.d.	0.01	n.d.	n.d.
14	25.79	1-Phenylethanone	95	120.15	C_8_H_8_O	98-86-2	n.d.	0.01	n.d.	n.d.
15	26.05	Octan-1-ol	94	130.23	C_8_H_18_O	111-87-5	n.d.	n.d.	n.d.	0.03
16	27.30	Non-1-en-4-ol	85	142.24	C_9_H_18_O	35192-73-5	n.d.	n.d.	0.03	0.01
17	27.58	Linalool	90	154.25	C_10_H_18_O	78-70-6	n.d.	n.d.	n.d.	0.01
18	27.78	Nonanal	92	142.24	C_9_H_18_O	124-19-6	0.03	n.d.	0.02	0.02
19	29.85	2,6,6-trimethyl-2-cyclohexene-1,4-dione	92	152.19	C_9_H_12_O_2_	1125-21-9	n.d.	n.d.	0.03	0.02
20	32.95	Decanal	91	156.26	C_10_H_20_O	112-31-2	n.d.	n.d.	0.02	0.01
21	33.64	2,6,6-trimethyl-1-cyclohexene-1-carboxaldehyde	83	152.23	C_10_H_16_O	4884-24-6	0.02	n.d.	n.d.	n.d.
22	35.67	2-(2,6,6-trimethylcyclohexen-1-yl) acetaldehyde	89	166.26	C_11_H_18_O	472-66-2	n.d.	n.d.	0.02	n.d.
23	35.80	Ethyl 5-methyl-3,4-dihydro-2H-pyran-4-carboxylate	83	170.21	C_9_H_14_O_3_	38858-64-9	n.d.	n.d.	0.05	n.d.
24	35.92	3,5,5-trimethyl-hex-1-ene	86	126.24	C_9_H_18_	4316-65-8	n.d.	0.02	0.29	0.03
25	36.18	Decan-1-ol	93	158.28	C_10_H_22_O	112-30-1	n.d.	0.01	0.09	0.36
26	36.58	Dec-1-en-3-one	92	154.22	C_10_H_18_O	56606-79-2	0.42	0.02	0.10	0.17
27	37.17	Undecan-2-one	95	170.29	C_11_H_22_O	112-12-9	n.d.	n.d.	0.14	0.02
28	37.80	Undecanal	96	170.29	C_11_H_22_O	112-44-7	n.d.	n.d.	n.d.	0.09
29	38.26	(2*E*,4*E*)-deca-2,4-dienal	94	152.23	C_10_H_16_O	25152-84-5	n.d.	n.d.	0.05	0.02
**30**	**38.37**	**Ethyl cyclohexanecarboxylate**	**87**	**156.22**	**C_9_H_16_O_2_**	** 3289-28-9 **	0.25	n.d.	**0.54**	0.07
31	38.76	Bicyclo(3.3.1)nonane-2,6-dione	83	152.19	C_9_H_12_O_2_	16473-11-3	0.17	n.d.	0.35	0.08
**32**	**39.00**	**(*R*)-5,7-dimethyl-1,6-octadiene (Isocitronellene)**	**87**	**138.25**	**C_10_H_18_**	** 85006-04-8 **	**0.88**	n.d.	0.04	0.03
33	39.42	Hexacosan-1-ol	81	382.7	C_26_H_54_O	506-52-5	n.d.	n.d.	0.30	n.d.
34	**40.20**	Eugenol	95	164.20	C_10_H_12_O_2_	97-53-0	0.19	n.d.	n.d.	n.d.
35	41.98	Nona-3,5-dien-2-one	84	138.21	C_9_H_14_O	80387-31-1	0.11	n.d.	0.45	0.16
36	42.18	6,10-dimethylundecan-2-one	95	198.34	C_12_H_26_O	1604-34-8	0.05	n.d.	0.32	0.09
**37**	**44.29**	**Geranylacetone**	**84**	**194.31**	**C_13_H_22_O**	** 689-67-8 **	0.14	n.d.	**0.70**	n.d.
38	42.37	Dodecanal	92	184.32	C_12_H_24_O	112-54-9	n.d.	n.d.	n.d.	0.05
39	43.40	(*E*)-4-[(1*R*,5*S*)-2,5,6,6-tetramethylcyclohex-2-en-1-yl]but-3-en-2-one	85	206.32	C_14_H_22_O	79-69-6	n.d.	n.d.	n.d.	0.15
40	44.08	3,7,11-trimethyldodecan-1-ol (hexahydrofarnesol)	92	228.41	C_15_H_32_O	6750-34-1	n.d.	0.14	n.d.	0.24
41	44.29	(*Z*)-6,10-dimethyl-5,9-undecadien-2-one	93	194.31	C_13_H_22_O	3879-26-3	n.d.	n.d.	n.d.	0.17
42	44.73	3,7,11-trimethyl-dodecan-1-ol	83	228.31	C_15_H_32_O	6750-34-1	0.32	0.07	0.18	0.29
**43**	45.08	(*Z*)-dec-3-enyl acetate	89	198.30	C_12_H_22_O_2_	81634-99-3	n.d.	n.d.	0.18	n.d.
**44**	**45.17**	**Dodecan-1-ol**	**94**	**186.33**	**C_12_H_26_O**	112-53-8	**1.06**	**1.02**	**1.03**	**3.65**
**45**	**45.91**	**(*E*)-4-(2,6,6-trimethyl-1-cyclohexen-1-yl)-3-buten-2-one**	**85**	**192.30**	**C_13_H_20_O**	14901-07-6	**2.76**	**0.53**	**5.41**	**4.34**
**46**	**46.07**	**Tridecan-2-one**	88	198.34	C_13_H_26_O	593-08-8	0.12	0.36	**0.67**	0.31
**47**	**46.22**	**Hexadecane**	**96**	**226.44**	**C_16_H_34_**	544-76-3	**0.58**	0.08	**0.84**	**1.30**
**48**	**46.34**	**Dimethyl 1,4-benzenedicarboxylate**	**88**	**194.18**	**C_10_H_10_O_4_**	120-61-6	n.d.	0.06	0.19	**1.17**
49	46.89	*n*-pentadecan-1-ol	94	228.41	C_15_H_32_O	629-76-5	n.d.	n.d.	0.02	0.23
50	47.33	(*E*)-Octadec-5-ene	91	252.47	C_18_H_36_	7206-21-5	n.d.	0.09	n.d.	n.d.
51	47.86	5,6,7,7a-Tetrahydro-4,4,7a-trimethyl-2(4H)-benzofuranone	90	180.24	C_11_H_16_O_2_	15356-74-8	0.27	n.d.	0.45	0.28
**52**	**48.31**	**(*Z*)-dodec-5-enoic acid**	95	198.30	C_11_H_22_O_2_	2430-94-6	n.d.	**2.79**	n.d.	n.d.
53	48.92	(*E*)-3,7,11-Trimethyl-1,6,10-dodecatrien-3-ol	89	222.36	C_15_H_26_O	40716-66-3	n.d.	n.d.	0.08	n.d.
**54**	**49.12**	**Dodecanoic acid (lauric acid)**	97	200.31	C_12_H_24_O_2_	143-07-7	n.d.	**3.78**	0.22	0.08
55	49.31	Tetradecan-1-ol	84	214.38	C_14_H_30_O	112-72-1	n.d.	0.26	0.37	0.31
56	49.90	2-O-(4-methylpentyl) 1-O-octadecyl oxalate	84	426.70	C_26_H_50_O_4_	29590-28-1	0.04	n.d.	0.11	0.05
57	50.16	Heptadecane	92	240.46	C_17_H_36_	629-78-7	0.08	n.d.	0.05	0.05
58	50.62	Tetradecanal	94	212.37	C_14_H_28_O	124-25-4	0.14	n.d.	0.08	0.20
59	51.26	2-methylhexadecan-1-ol	90	256.46	C_17_H_36_O	2490-48-4	n.d.	0.35	0.18	0.39
60	51.42	Diphenylmethanone	95	182.21	C_13_H_10_O	119-61-9	0.34	0.29	0.39	n.d.
61	52.16	2-ethyldodecan-1-ol	88	214.38	C_14_H_30_O	19780-33-7	0.24	n.d.	0.23	0.18
**62**	**52.30**	**alpha-Cadinol**	**87**	**222.36**	**C_15_H_26_O**	**481-34-5**	**1.24**	0.12	n.d.	n.d.
**63**	**52.65**	**Hexadecan-1-ol**	**92**	**242.44**	**C_16_H_34_O**	**36653-82-4**	**1.56**	**2.60**	**7.20**	**7.99**
64	52.91	(*E*)-heptadec-8-ene	77	238.45	C_17_H_34_	54290-12-9	n.d.	n.d.	0.11	0.09
**65**	**53.03**	**Pentadec-1-ene**	92	210.40	C_15_H_30_	13360-61-7	n.d.	n.d.	0.14	**2.62**
**66**	**53.22**	**11-pentan-3-ylhenicosane**	**89**	**366.70**	**C_26_H_54_**	**55282-11-6**	**0.65**	0.43	**1.11**	**1.41**
67	53.38	2-tetradecoxyethanol	81	258.44	C_16_H_34_O_2_	2136-70-1	0.24	0.32	0.44	0.37
**68**	**53.64**	**Tridecanal**	**93**	**198.34**	**C_13_H_26_O**	**10486-19-8**	**0.60**	0.37	**0.81**	**0.43**
69	53.72	2-hexadecoxyethanol	87	286.49	C_18_H_38_O_2_	2136-71-2	0.25	n.d.	n.d.	n.d.
**70**	**54.12**	**2-octadecoxyethanol**	**90**	**314.54**	**C_20_H_42_O_2_**	**2136-72-3**	n.d.	0.42	0.41	**0.50**
71	54.30	*n*-Nonadecan-1-ol	88	284.52	C_19_H_40_O	1454-84-8	n.d.	0.11	n.d.	n.d.
72	54.52	(*E*)-icos-3-ene	86	280.53	C_20_H_40_	74685-33-9	n.d.	0.14	n.d.	n.d.
**73**	**54.99**	**Myristic acid**	**97**	**228.37**	**C_14_H_28_O_2_**	**544-63-8**	n.d.	**7.60**	0.31	0.18
**74**	**55.22**	***n*-Pentadecanol**	**93**	**228.41**	**C_15_H_32_O**	**629-76-5**	0.46	**0.85**	**0.72**	**1.19**
**75**	**55.63**	**Eicosanoic acid**	**87**	**312.53**	**C_20_H_40_O_2_**	**506-30-9**	**2.58**	**0.51**	**1.14**	**n.d.**
76	55.69	(*Z*)-Undec-4-enal	87	168.27	C_11_H_20_O	68820-32-6	0.36	n.d.	n.d.	n.d.
77	56.17	Isopropyl myristate	93	270.45	C_17_H_34_O_2_	110-27-0	0.30	n.d.	n.d.	n.d.
**78**	**56.63**	**6,10,14-Trimethyl-2-pentadecanone**	**96**	**268.5**	**C_18_H_36_O**	**502-69-2**	**2.99**	**0.75**	**5.98**	**5.72**
**79**	**56.89**	**2,3-diisopropyl-naphthalene**	**77**	**212.33**	**C_16_H_20_**	**94133-81-0**	**1.47**	0.36	**0.55**	0.42
**80**	**56.98**	**Oleyl alcohol**	93	268.5	C_18_H_36_O	143-28-2	n.d.	**0.68**	**4.41**	**5.96**
**81**	**57.18**	**bis(2-methylpropyl) benzene-1,2-dicarboxylate**	**97**	**278.34**	**C_16_H_22_O_4_**	**84-69-5**	**2.11**	**0.51**	**1.11**	n.d.
**82**	**57.52**	**Nonadec-1-ene**	**93**	**266.50**	**C_19_H_38_**	**18435-45-5**	n.d.	n.d.	0.20	**1.30**
**83**	**58.11**	**Farnesyl acetone**	**90**	**266.43**	**C_18_H_30_O**	**1117-52-8**	**0.93**	**0.57**	**1.28**	**1.05**
**84**	**58.62**	**Palmitoleic acid**	**95**	**254.40**	**C_16_H_30_O_2_**	**373-49-9**	**2.79**	**11.94**	n.d.	n.d.
**85**	**58.90**	**Palmitic acid**	**93**	**256.42**	**C_16_H_32_O_2_**	**57-10-3**	**40.15**	**31.92**	**26.81**	**18.62**
**86**	**60.14**	**Arachidonic acid**	**90**	**304.46**	**C_20_H_32_O_2_**	**506-32-1**	**0.96**	**1.45**	0.14	0.27
**87**	**60.30**	**Methyl Arachidonate**	**83**	**318.49**	**C_21_H_34_O_2_**	**2566-89-4**	**4.00**	**2.49**	**1.55**	**1.74**
**88**	**60.81**	***n*-Nonadecan-1-ol**	**89**	**284.52**	**C_19_H_40_O**	**1454-84-8**	**1.67**	**3.13**	**4.13**	**4.34**
**89**	**60.84**	**(*Z*,*Z*)-2,13-Octadecadien-1-ol**	85	266.46	C_18_H_34_	123551-47-3	0.21	n.d.	**1.67**	n.d.
**90**	**61.21**	**5-dodecyloxolan-2-one**	**90**	**254.41**	**C_16_H_30_O_2_**	**730-46-1**	**1.44**	0.21	**1.94**	**0.88**
**91**	**61.29**	**Phytol**	**96**	**296.53**	**C_20_H_40_O**	**150-86-7**	**3.97**	**2.90**	**5.76**	**14.20**
**92**	**61.69**	**Oleic acid**	91	282.46	C_18_H_34_O_2_	112-80-1	0.23	**1.63**	0.12	0.29
93	61.81	Heptadecyl heptadecanoate	81	508.92	C_34_H_68_O_2_	n.a.	0.22	n.d.	0.12	0.40
94	61.99	Ascorbyl palmitate	70	414.53	C_22_H_38_O_7_	137-66-6	0.32	n.d.	n.d.	0.12
95	62.39	Octadecyl propan-2-yl sulphite	82	376.60	C_21_H_44_O_3_S	n.a.	0.22	n.d.	n.d.	0.19
**96**	**62.58**	**Tetradecyl benzoate**	**78**	**318.50**	**C_21_H_34_O_2_**	**70682-72-3**	**1.10**	**4.37**	**0.53**	0.09
**97**	**63.04**	**Pentadecyl benzoate**	80	332.50	C_22_H_36_O_2_	n.a.	0.07	**0.50**	0.10	0.04
**98**	**63.98**	**Tridecyl benzoate**	**88**	304.50	C_20_H_32_O_2_	29376-83-8	0.16	**1.28**	0.40	0.05
**99**	**64.22**	**2-(Octadecyloxy)ethanol**	**91**	**314.55**	**C_20_H_42_O_2_**	**9005-00-9**	**0.28**	**1.04**	**0.76**	**1.46**
100	64.39	Stearic acid	80	284.48	C_18_H_36_O_2_	57-11-4	0.36	0.09	n.d.	n.d.
101	64.96	1-Decylsulfonyldecane	79	346.61	C_20_H_42_O_2_S	111530-37-1	0.13	n.d.	n.d.	0.14
102	66.14	Icosane	94	282.55	C_20_H_42_	112-95-8	0.16	n.d.	0.30	0.11
103	67.28	Nonacosane	93	408.79	C_29_H_60_	630-03-5	0.34	0.05	0.09	0.14
104	70.37	Squalene	90	410.73	C_30_H_50_	111-02-4	0.18	0.04	0.20	0.07
		**TOTAL IDENTIFIED COMPOUNDS (from peak total area)**					**84.37**	**89.43**	**85.44**	**87.41**

Compounds detected in amounts higher than 0.5% are written in bold; n.d.—not detected; n.a.—not available.

## Data Availability

Data is contained within the article.

## Sample Availability

Samples of the EOs are not available from the authors.
